# Adverse Events as a Surrogate of Sufficient Pharmacological Exposure in Metronomic Combination Chemotherapy: Extended Real-World Cohort Analysis of the FulVEC Regimen in Metastatic ER+/HER2− Breast Cancer

**DOI:** 10.3390/cancers18142303

**Published:** 2026-07-17

**Authors:** Anna Buda-Nowak, Maciej Lubaś, Michał Jurczyk, Łukasz Kwinta, Anna Michałowska-Kaczmarczyk, Agnieszka Przywara-Sikora, Kamil Konopka, Maciej Koniewski, Joanna Kadłuczka, Olga Szczerbak, Piotr J. Wysocki

**Affiliations:** 1 Department of Oncology, Jagiellonian University-Medical College, 31-501 Krakow, Poland; abuda@su.krakow.pl (A.B.-N.); lkwinta@su.krakow.pl (Ł.K.); kkonopka@su.krakow.pl (K.K.); 2 Department of Oncology, University Hospital in Krakow, 31-501 Krakow, Poland; mlubas@su.krakow.pl (M.L.); mijurczyk@su.krakow.pl (M.J.); 3 Department of Pathophysiology, Jagiellonian University-Medical College, 31-008 Krakow, Poland; 4 Institute of Sociology, Jagiellonian University, 31-007 Krakow, Poland; 5 Student Scientific Society, Jagiellonian University-Medical College, 31-008 Krakow, Poland

**Keywords:** breast cancer, metronomic chemotherapy, chemo-endocrine therapy, FulVEC, dose titration, pharmacological exposure, adverse events, CDK4/6 inhibitor resistance

## Abstract

Background: Metronomic chemo-endocrine therapy combining fulvestrant with metronomic VEC (vinorelbine, cyclophosphamide, and capecitabine)—the FulVEC regimen—demonstrated promising activity in an initial cohort of 38 patients with advanced ER+/HER2− breast cancer (JCM 2023). Here, we present an extended analysis of 72 consecutive patients, with a focus on a novel hypothesis: that treatment-emergent adverse events (AEs) requiring dose modification serve as a surrogate for sufficient pharmacological exposure in metronomic combination chemotherapy. Methods: Retrospective analysis of 72 consecutive patients with metastatic ER+/HER2− breast cancer treated with FulVEC at Jagiellonian University Hospital between 2018 and 2024. Efficacy endpoints included progression-free survival (PFS), overall survival (OS), and biochemical response, as assessed by CA15-3 dynamics. Patients were stratified by AE severity requiring intervention (grade 0: no modification; grade 1: dose reduction; and grade 2: treatment delay). The association between AE grade and efficacy outcomes was assessed using Spearman’s correlation, the log-rank test, and the chi-square test. Results: The median PFS was 8.5 months, and the median OS was 18.0 months. The biochemical benefit rate (any CA15-3 decline) was 81.6%. No statistically significant differences in efficacy were observed according to prior exposure to CDK4/6 inhibitors, fulvestrant, or cytotoxic components of the FulVEC regimen. A monotonic dose–response relationship was observed across AE grade categories: non-progression rates increased from 73.2% (grade 0) to 84.2% (grade 1) and 91.7% (grade 2); biochemical benefit rates from 68.4% to 90.9% and 100.0%; and median CA15-3 reduction deepened from −34% to −44% and −52%, respectively (Spearman r = 0.258 and *p* = 0.043 for AE grade vs. treatment duration). Formal log-rank comparisons of PFS and OS across the three AE-grade categories did not reach statistical significance (*p* = 0.583 and *p* = 0.743, respectively), reflecting the limited size of the treatment-delay subgroup (n = 12); the dose–response signal should, therefore, be regarded as exploratory. No patient required permanent treatment discontinuation due to toxicity. Conclusions: The extended FulVEC cohort confirms durable activity and a reproducible, manageable safety profile in a heavily pretreated population, including CDK4/6i-refractory patients. The exploratory, hypothesis-generating observation of a dose–response gradient between AE severity and clinical outcomes raises the possibility that treatment-emergent AEs may, in some patients, reflect adequate pharmacological exposure to the metronomic regimen. Given confounding by treatment duration and survivor bias, and the absence of pharmacokinetic data, this hypothesis requires prospective validation and does not, at this stage, support any change to current treatment practice.

## Simple Summary

The typical clinical scenario in advanced ER+/HER2− breast cancer of resistance development to endocrine therapy and CDK4/6 inhibitors represents one of the most difficult challenges in oncology. After progression on these agents, available treatment options are limited and typically provide only a few months of disease control. In this study, we report the outcomes of 72 patients with advanced ER+/HER2− breast cancer treated with FulVEC. This four-drug metronomic regimen combines fulvestrant with three oral cytotoxic agents (vinorelbine, cyclophosphamide, and capecitabine) administered continuously at low doses. Despite the majority of patients having previously received CDK4/6 inhibitors, fulvestrant, and, in many cases, cytotoxic chemotherapy, FulVEC achieved a median time without disease progression of 8.5 months and a median overall survival of 18.0 months, which compares favorably with available alternatives in this refractory setting. We also report an exploratory observation suggesting that patients who require dose adjustments due to treatment-related adverse events may derive greater clinical benefit, which we interpret as evidence that adequate drug exposure is critical for the efficacy of metronomic chemotherapy. This finding supports the concept of individually tailored dose escalation in metronomic combination regimens, which warrants prospective clinical evaluation.

## 1. Introduction

Endocrine therapy (ET) combined with CDK4/6 inhibitors represents the standard first-line treatment for metastatic hormone receptor-positive, HER2-non-overexpressing (ER+/HER2−) breast cancer. Despite high initial response rates, disease progression inevitably occurs, leaving patients in urgent need of further systemic treatment options. The post-CDK4/6i setting is clinically challenging: rechallenge with CDK4/6 inhibitors yields a median PFS of 3.7–4.8 months [1,2], while antibody–drug conjugates (ADCs), though highly active, are associated with substantial toxicity—including interstitial lung disease, peripheral neuropathy, and ocular toxicity—rendering long-term administration challenging and potentially detrimental to quality of life, in addition to remaining inaccessible in many healthcare systems due to regulatory and cost constraints.

Metronomic chemotherapy (MCT), which involves continuous administration of cytotoxic agents at low, sub-maximum-tolerated doses (sub-MTDs), is an attractive option in this context. Through antiangiogenic, immunomodulatory, and direct antiproliferative mechanisms, MCT may circumvent classical resistance pathways while maintaining an acceptable safety profile [3,4,5]. Beyond breast cancer, the activity of MCT has been demonstrated across multiple tumor types, including gastrointestinal cancers, prostate cancer, ovarian cancer, and cutaneous malignancies, supporting the view that metronomic scheduling unlocks tumor-agnostic pharmacological properties distinct from those of conventional chemotherapy administered at the maximum tolerated dose (MTD) [6,7,8]. The combination of MCT with endocrine agents (chemo-endocrine therapy (CET)) further leverages potential synergism between the hormonal and cytotoxic components [9,10]. This rationale has now been validated in a phase III setting: the MECCA trial demonstrated that metronomic capecitabine combined with an aromatase inhibitor significantly prolonged PFS to 20.9 months compared with 11.9 months for the aromatase inhibitor alone in advanced ER+/HER2− breast cancer [11], establishing metronomic CET as a legitimate first-line strategy and providing the strongest prospective evidence to date for this treatment paradigm [12].

We previously reported the results of the FulVEC regimen—fulvestrant combined with metronomic VEC (40 mg of vinorelbine three times weekly, 50 mg of cyclophosphamide daily, and 500 mg of capecitabine three times daily)—in 38 consecutive patients with advanced ER+/HER2− breast cancer, demonstrating a median PFS of 8.4 months and an OS of 21.5 months, with preserved efficacy irrespective of prior CDK4/6i or fulvestrant exposure [13]. Here, we present an expanded real-world cohort of 72 patients, enabling more reliable subgroup analyses and, critically, allowing us to examine a novel hypothesis derived from clinical observation formally.

A central unresolved issue in metronomic chemotherapy is the definition of the optimal biological dose. Unlike conventional MTD chemotherapy, where dose reduction unambiguously reduces efficacy, metronomic regimens are designed to operate at low exposure levels. However, inter-individual pharmacokinetic variability means that a population-level fixed dose may underdose a substantial proportion of patients. In single-agent chemotherapy, the lack of dose titration options forces clinicians to accept this variability. Crucially, in multi-drug metronomic platforms such as FulVEC, the four-component structure provides multiple pharmacodynamic axes that can be independently adjusted in response to organ-specific toxicity signals. No validated pharmacodynamic biomarker currently exists to identify, at the individual-patient level, whether a fixed metronomic dose has achieved adequate biological exposure. Whether treatment-emergent toxicity, which represents an inexpensive, universally available clinical observation, could serve as such an indicator has not, to our knowledge, been previously examined and represents the specific knowledge gap addressed by the present analysis.

This extended cohort analysis was designed with two specific aims: (i) to confirm, in a larger population, the efficacy and safety of FulVEC previously reported in 38 patients, and (ii) to explore, in a purely hypothesis-generating manner, whether the occurrence and severity of treatment-emergent AEs are associated with measures of clinical benefit, consistent with the hypothesis that AEs may, in some patients, reflect sufficient pharmacological exposure. We emphasize from the outset that this second aim is exploratory; no causal or diagnostic claim is intended, and confirmation would require a dedicated prospective study with pharmacokinetic sampling.

## 2. Materials and Methods

### 2.1. Patients

We retrospectively collected data on consecutive patients with advanced metastatic ER+/HER2− breast cancer treated with the FulVEC metronomic chemo-endocrine regimen at the Department of Oncology, Jagiellonian University Hospital, Krakow, Poland, between May 2018 and December 2024. The inclusion criteria were histologically confirmed advanced (metastatic or locally recurrent inoperable) breast cancer; expression of estrogen receptor (ER) and non-overexpression of HER2; an ECOG performance status of 0–3; and prior failure of at least one line of systemic treatment, which must have been based on endocrine therapy. This study was conducted in accordance with the Declaration of Helsinki and approved by the Institutional Ethics Committee of Jagiellonian University (approval no. 1072.6120.229.2022).

### 2.2. Treatment

The FulVEC regimen consisted of 500 mg of fulvestrant intramuscularly on days 1, 14, and 28, and monthly thereafter; 40 mg of vinorelbine orally three times weekly; 50 mg of cyclophosphamide orally once daily; and 500 mg of capecitabine orally three times daily. All premenopausal patients received concurrent gonadotropin-releasing hormone analog therapy to achieve postmenopausal estrogen levels before fulvestrant initiation.

Dose adjustments were performed in a stepwise, drug-specific manner based on treatment-emergent AEs. Vinorelbine-related AEs led to a reduction from 40 mg three times weekly to 30 mg every other day. Capecitabine-related AEs (primarily hand–foot syndrome) led to sequential reductions from 500 mg three times daily to 500 mg twice daily, and then once daily. Cyclophosphamide-related AEs led to a reduction in frequency from daily to every other day. Treatment was withheld temporarily in the event of grade ≥ 3 AEs until resolution, after which it was resumed at a reduced dose.

### 2.3. Efficacy Endpoints

The primary endpoints were PFS and OS. PFS was defined as the time from FulVEC initiation to radiographic or clinical progression, or death from any cause. OS was defined as the time from FulVEC initiation to death from any cause. Biochemical response was assessed in patients with evaluable CA15-3 at baseline and during treatment. The biochemical response rate was defined as a ≥50% serum CA15-3 reduction from baseline; biochemical stabilization as a 1–49% reduction; biochemical benefit as any CA15-3 decline; biochemical progression as any increase in CA15-3; and non-biochemical progression as no increase in CA15-3.

### 2.4. AE Grade Classification and Dose–Response Analysis

For the exploratory dose–response analysis, patients were categorized into three AE exposure grades: grade 0 (no AEs requiring any modification); grade 1 (AEs requiring dose reduction of at least one FulVEC component); and grade 2 (AEs requiring temporary treatment delay in addition to or instead of dose reduction). This ordinal classification was used as a proxy for achieved pharmacological exposure. Importantly, this classification is based solely on the clinical intervention performed (dose reduction versus treatment delay) and does not correspond to formal CTCAE (Common Terminology Criteria for Adverse Events) severity grading. It, therefore, does not capture the specific organ system, causality assessment, or CTCAE grade of individual toxicities, and should not be interpreted as, or compared with, a standard AE grading system. The association between AE grade and efficacy endpoints was assessed using Spearman’s rank correlation, the Kruskal–Wallis test, the chi-square test, and Kaplan–Meier survival analysis with the log-rank test.

### 2.5. Statistical Analysis

Survival distributions were estimated using the Kaplan–Meier method and compared using the log-rank test. Hazard ratios were estimated using the Cox proportional hazards model with Jackknife standard errors. Associations between categorical variables were assessed using the chi-square test or Fisher’s exact test as appropriate. The normality of continuous variables (treatment duration; CA15-3 percentage change) was assessed using the Shapiro–Wilk test; both variables departed significantly from a normal distribution (*p* < 0.05), justifying the use of non-parametric methods. Spearman’s rank correlation was used for ordinal and non-normally distributed continuous variables. All tests were two-sided; *p* < 0.05 was considered statistically significant. Due to the exploratory nature of the AE-grade dose–response analysis, *p*-values between 0.05 and 0.10 are reported as trends. Statistical analyses were performed using Stata/MP 17 and Python coding.

## 3. Results

### 3.1. Patient Characteristics

Between May 2018 and December 2024, 72 consecutive patients with advanced ER+/HER2− breast cancer received the FulVEC regimen. Detailed patient characteristics are presented in Table 1. The median age was 50 years (range: 31–79). All patients had disseminated disease; the most common metastatic sites were bone, liver, and lung. The median number of prior systemic treatment lines was two; 46% of patients had received ≥3 prior lines. Notably, 62% of patients had been previously treated with CDK4/6 inhibitors and 56% with fulvestrant, indicating a more heavily pretreated population than in our initial report. Prior exposure to at least one cytotoxic component of the FulVEC regimen (vinorelbine, cyclophosphamide, or capecitabine) was documented in 46% of patients.

### 3.2. Efficacy

**Figure 1:**
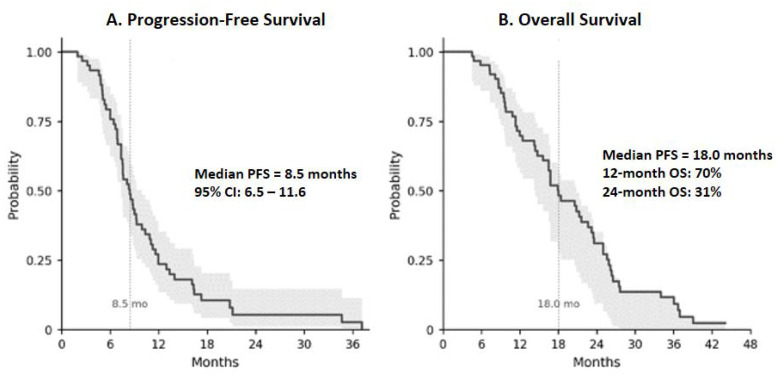
Kaplan–Meier survival curves for the FulVEC cohort (N = 72). (**A**) Progression-free survival: median: 8.5 months (95% CI: 6.5–11.6); 12-month rate: 23%; and 24-month rate: 5%. (**B**) Overall survival: median: 18.0 months; 12-month rate: 70%; and 24-month rate: 31%. Shaded areas represent 95% confidence intervals.

The median follow-up was 24 months. The median PFS was 8.5 months (95% CI: 6.5–11.6) with 12- and 24-month PFS rates of 23% and 5%, respectively. The median OS was 18.0 months with 12- and 24-month OS rates of 70% and 31%, respectively (Table 2; Figure 1). Biochemical evaluation was available in 38 of 72 patients (53%). Biochemical benefit (any CA15-3 decline) was observed in 81.6% of evaluable patients; biochemical response (≥50% decline) in 44.7%; and biochemical stabilization in 36.8%. Biochemical progression (defined as any increase in CA15-3) occurred in 18.4% of evaluable patients.

**Figure 2:**
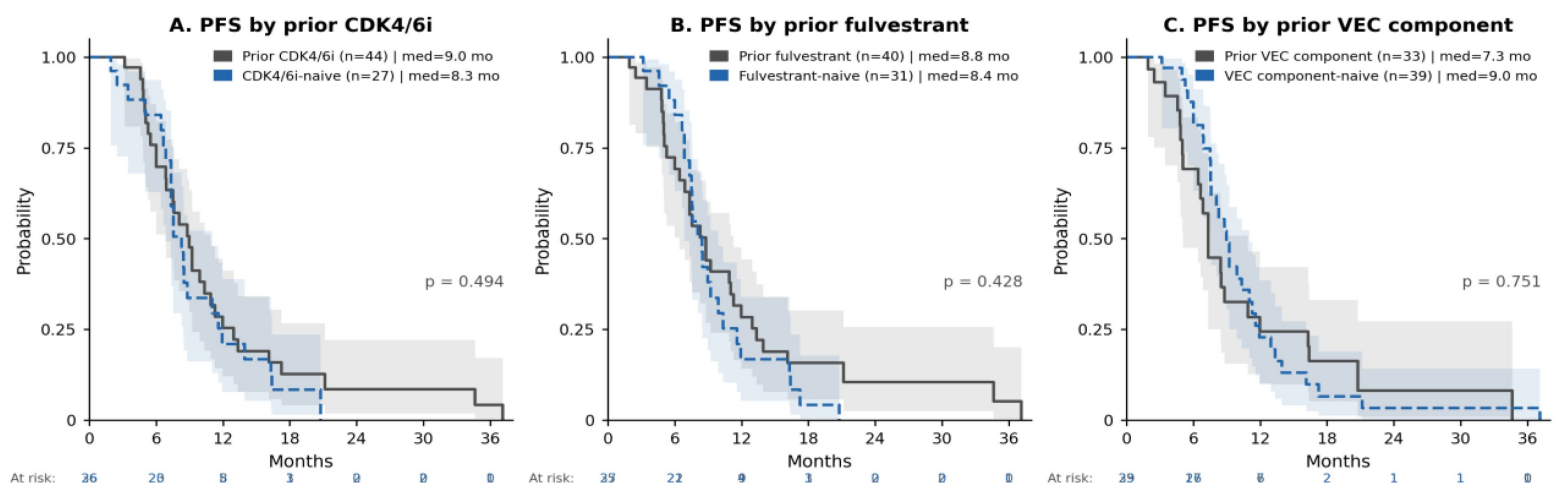
Progression-free survival by prior treatment exposure. (**A**) Prior CDK4/6 inhibitor (n = 44) vs. CDK4/6i-naive (n = 27); median: 9.0 vs. 8.3 months; log-rank *p* = 0.494. (**B**) Prior fulvestrant (n = 40) vs. fulvestrant-naive (n = 31); median: 8.8 vs. 8.5 months; log-rank *p* = 0.428. (**C**) Prior cytotoxic VEC component (n = 33) vs. VEC-naive (n = 39); median: 7.3 vs. 9.0 months; log-rank *p* = 0.751. Shaded areas represent 95% confidence intervals.

No statistically significant differences in PFS or OS were observed between patients with and without prior CDK4/6 inhibitor therapy (PFS: 9.0 vs. 8.3 months; log-rank *p* = 0.494), prior fulvestrant exposure (PFS: 8.8 vs. 8.5 months; *p* = 0.428), or prior exposure to cytotoxic VEC components (PFS: 7.3 vs. 9.0 months; *p* = 0.751) [Figure 2].

### 3.3. Toxicity-Guided Dose Modification and Exploratory Dose–Response Analysis

Any treatment modification (dose reduction and/or treatment delay) was required in 31 of 72 patients (43%). Dose reduction of at least one FulVEC component was performed in 29 patients (41%); treatment delay was performed in 12 patients (17%), including nine patients (13%) in whom delay was directly attributable to AEs. No patient required permanent treatment discontinuation due to FulVEC-related toxicity.

**Figure 3:**
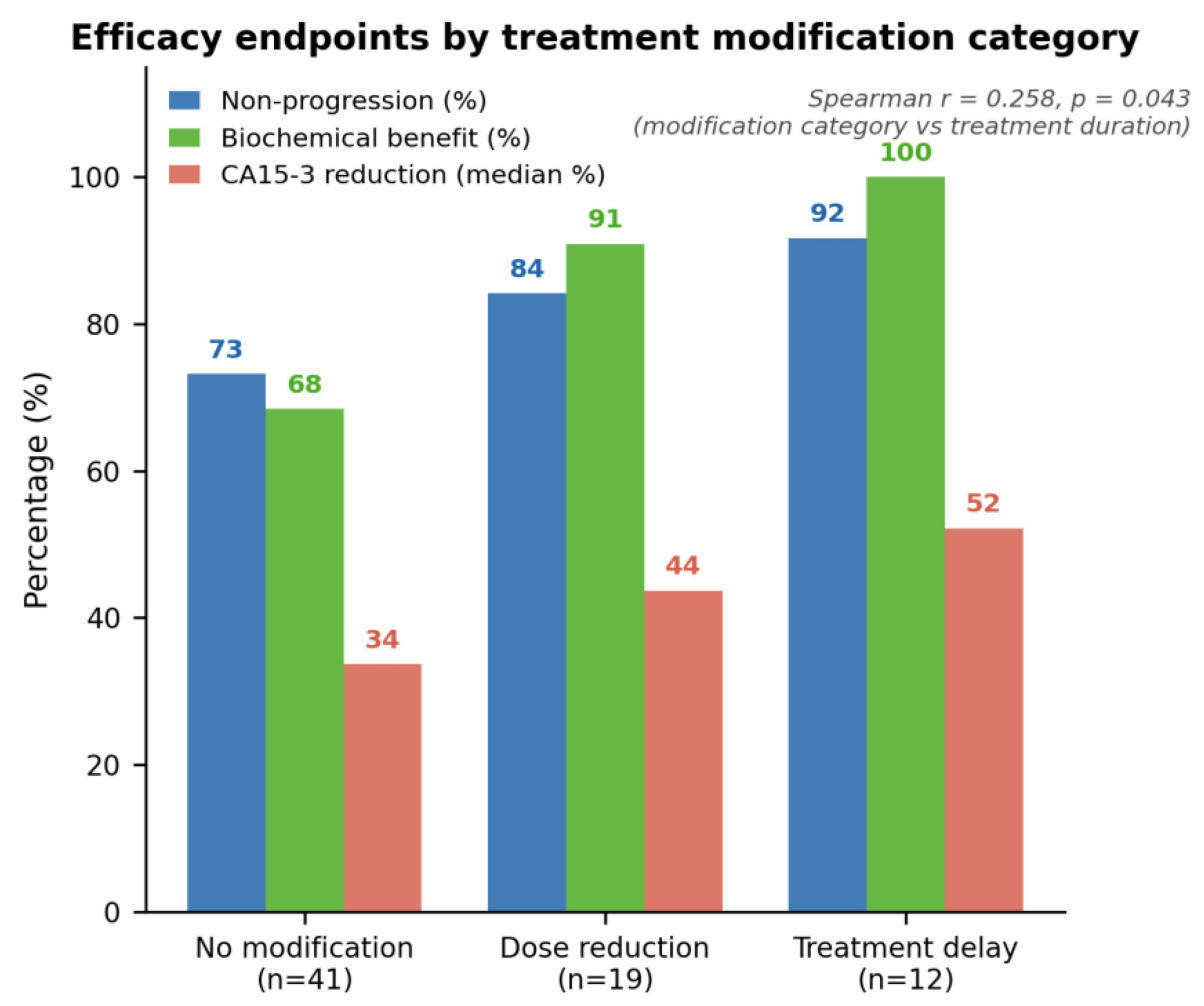
Efficacy outcomes by treatment modification status. Non-progression rate, biochemical benefit rate (any CA15-3 decline), and median CA15-3 reduction from baseline are shown for patients with no dose modification required (n = 41), dose reduction of at least one FulVEC component (n = 19), and treatment delay (n = 12). All three endpoints improved progressively as the intensity of treatment modification increased. Spearman correlation of modification category with treatment duration: r = 0.258; *p* = 0.043.

According to the predefined AE-grade categories, grade 0 (no modification required) was observed in 41 patients, grade 1 (dose reduction only) in 19 patients, and grade 2 (treatment delay with or without dose reduction) in 12 patients. A monotonic dose–response relationship was observed across all efficacy endpoints (Table 3; Figure 3): non-biochemical progression rates (percentage of patients without any increase of CA15-3) increased from 73.2% (grade 0) to 84.2% (grade 1) and 91.7% (grade 2); biochemical benefit rates from 68.4% to 90.9% and 100.0%; and median CA15-3 reduction deepened from −34% to −44% and −52%, respectively. Spearman’s correlation between AE grade and treatment duration (a surrogate of ongoing disease control) was r = 0.258 (*p* = 0.043). In comparison, formal log-rank comparisons of PFS and OS across the three groups did not reach statistical significance (*p* = 0.583 and *p* = 0.743, respectively).

### 3.4. Safety

Dose reductions were required in 41% of patients, most commonly due to myelotoxicity (primarily neutropenia) or hand–foot syndrome (HFS). Treatment delays occurred in 17% of patients. The safety outcomes are summarized in Table 4. Because this was a retrospective analysis spanning six years of clinical practice, individual adverse events were not systematically coded by specific type or CTCAE severity grade in the source dataset; only the resulting clinical intervention (dose reduction or treatment delay) and its broadly attributed cause (myelotoxicity-related versus hand–foot-syndrome-related) were consistently documented (Table 4).

## 4. Discussion

The extended FulVEC cohort of 72 consecutive patients confirms and consolidates the findings of our initial report. The near-identical median PFS (8.5 vs. 8.4 months), despite the higher proportion of CDK4/6i-pretreated patients (62% vs. 47%), suggests that FulVEC activity is not compromised by prior CDK4/6i exposure. This finding has direct clinical relevance as CDK4/6i-refractory disease becomes the predominant treatment context in advanced ER+/HER2− breast cancer. The safety profile was likewise reproducible: dose reductions in 41% vs. 46%, treatment delays in 17% vs. 23%, and no permanent treatment discontinuation due to toxicity in either cohort.

In the PACE trial, continuation of palbociclib beyond CDK4/6i progression offered no benefit: the median PFS was 4.8 months with fulvestrant alone versus 4.6 months with fulvestrant plus palbociclib (HR 1.11; *p* = 0.62) [1]. Notably, the triplet of fulvestrant, palbociclib, and the PD-L1 inhibitor avelumab achieved a median PFS of 8.1 months [1], numerically similar to FulVEC. However, since this signal did not reach statistical significance (*p* = 0.23), its interpretation requires caution; the activity of immune checkpoint inhibitors in ER+/HER2− breast cancer remains highly controversial. Multiple randomized trials consistently failed to demonstrate a meaningful benefit of PD-1/PD-L1 inhibition [14,15], leading to the luminal subtype being deemed among the least immunogenic solid tumors. A dedicated phase II study of fulvestrant plus palbociclib after prior palbociclib showed a median PFS of only 3.7 months [2]. Against this background, the 8.5-month PFS observed with FulVEC in a more heavily pretreated population is clinically meaningful.

Contextualizing FulVEC outcomes requires attention to population differences. Among the pivotal CDK4/6 inhibitor trials in aromatase inhibitor/tamoxifen-resistant ER+/HER2− breast cancer patients, only PALOMA-3 permitted prior chemotherapy for metastatic disease (up to one line, received by ~34% of patients), making it the sole trial with partial comparability to the present cohort [16]. MONARCH-2 [17] and MONALEESA-3 [18] enrolled only chemotherapy-naïve patients. In PALOMA-3, patients naive to fulvestrant and CDK4/6 inhibitors achieved a median PFS of 4.6 months with fulvestrant monotherapy and 9.5 months with palbociclib plus fulvestrant. Despite substantially heavier pretreatment (62% prior to CDK4/6i, 56% prior to fulvestrant, and 46% prior to any VEC component), FulVEC achieved a median PFS of 8.5 months, nearly double that of the PALOMA-3 control arm, and within the range reported for its active (palbociclib-containing) arm. We emphasize that this comparison is descriptive and hypothesis-generating only: PALOMA-3, PACE, and the real-world series cited below differ from the present cohort in study design, line of therapy, patient selection, and endpoint definitions, and no formal statistical comparison across studies is implied or possible. Real-world data from patients progressing on the AI plus CDK4/6i sequence are more directly relevant: fulvestrant monotherapy after CDK4/6i yields a median PFS of only 3.2 months [19,20]; chemotherapy-based regimens achieve 3.0 months PFS and 8.3 months OS [21]; and second-line PFS is 4.2–5.3 months with only 31% of patients reaching a third line [22]. FulVEC numerically compares favorably; however, given the retrospective, non-randomized nature of both the present cohort and the cited comparator series, these figures should be viewed as descriptive context rather than as evidence of superiority.

The biochemical benefit rate of 81.6% provides evidence of biological activity across the majority of patients. The independence of FulVEC efficacy from prior exposure to individual cytotoxic VEC components suggests that prior single-agent use does not confer cross-resistance to the multi-drug metronomic platform, most likely because continuous low-dose combination therapy operates through fundamentally different mechanisms than the same agents at MTD. This is further supported by the METEORA-II trial, which established the VEC backbone as an active metronomic platform superior to weekly paclitaxel in ER-positive, HER2-negative metastatic breast cancer [23]. Additional evidence for the activity of metronomic CET combinations in ER+/HER2− breast cancer comes from earlier phase II studies of fulvestrant-based metronomic regimens, including oral cyclophosphamide plus methotrexate combined with fulvestrant [24] and capecitabine-based chemo-endocrine combinations [25], which established proof-of-concept for this treatment approach prior to the development of the FulVEC platform. Given the modest size of the prior-treatment subgroups analyzed (Section 3.2), this study is likely underpowered to detect moderate differences in efficacy; these findings should, therefore, be interpreted as an absence of a demonstrated difference rather than confirmed equivalence of efficacy across prior-treatment subgroups.

The central exploratory finding of this analysis is an association between treatment modification (used as a proxy for AE severity) and efficacy outcomes: a directionally consistent gradient was observed across non-progression rates (73–92%), biochemical benefit (68–100%), and CA15-3 reduction (−34 to −52%), together with a statistically significant correlation with treatment duration (Spearman r = 0.258; *p* = 0.043). However, formal log-rank comparisons of PFS and OS across AE-grade categories did not reach significance (*p* = 0.583 and 0.743), and the treatment-delay subgroup comprised only 12 patients; this association must, therefore, be interpreted with considerable caution.

Several alternative, non-mutually exclusive explanations for this association must be considered before invoking a pharmacological exposure mechanism. First, the association may be confounded by treatment duration itself and by survivor bias: because dose modifications can only be recorded in patients who remained on treatment long enough to develop and report an AE, and patients with early progression or early death have, by definition, less opportunity to be classified as grade 1 or 2. This phenomenon may generate an apparent association between higher AE grade and longer survival independent of any true pharmacodynamic effect. This mechanism is, indeed, detectable in our data: all five patients treated for ≤90 days were classified as grade 0, confirming that very short exposure precludes AE-driven dose modification. However, this pattern cannot fully account for the observed gradient, as it involved only a small minority of the grade-0 subgroup (5 of 41 patients; 12%), and the median treatment duration in the grade-0 group was 223 days (~7.3 months), which was substantially longer than the minimum exposure required to develop treatment-emergent AEs in the grade-1 and grade-2 groups (minimum: 153–167 days). Survivor bias, therefore, likely contributes to, but does not appear sufficient to fully explain, the observed association. Second, the absence of AEs is not necessarily equivalent to inadequate drug exposure: some patients may be adequately dosed but simply tolerate therapy well due to favorable pharmacogenomic or metabolic profiles, while others may discontinue or reduce treatment early for reasons unrelated to cumulative exposure (e.g., patient preference, comorbidity, or logistic factors). We, therefore, explicitly avoid the categorical claim that absence of AEs identifies underdosed patients; at most, our data are compatible with, but do not establish, the hypothesis that AE occurrence may, in a subset of patients, reflect sufficient pharmacological exposure.

Critically, we did not measure plasma or intracellular drug concentrations and, therefore, cannot directly test whether AE occurrence correlates with pharmacokinetic exposure; the pharmacological exposure interpretation offered here is a hypothesis suggested by the pattern of clinical association, not a mechanism demonstrated by this study. With this caveat, we speculatively note that, in a four-component platform such as FulVEC, the organ-specific AE profile (e.g., hand–foot syndrome/diarrhea for capecitabine; neuropathy/abdominal discomfort for vinorelbine; and cystitis for cyclophosphamide) could, in principle, allow differential dose adjustment of individual components rather than uniform dose reduction (a feature not available with single-agent MCT). We refer to this as a working hypothesis of toxicity-guided adaptive dosing, to be tested prospectively; we do not propose it as a recommendation for current clinical practice, and a retrospective single-center cohort of this size is insufficient to support such a change.

**Figure 4:**
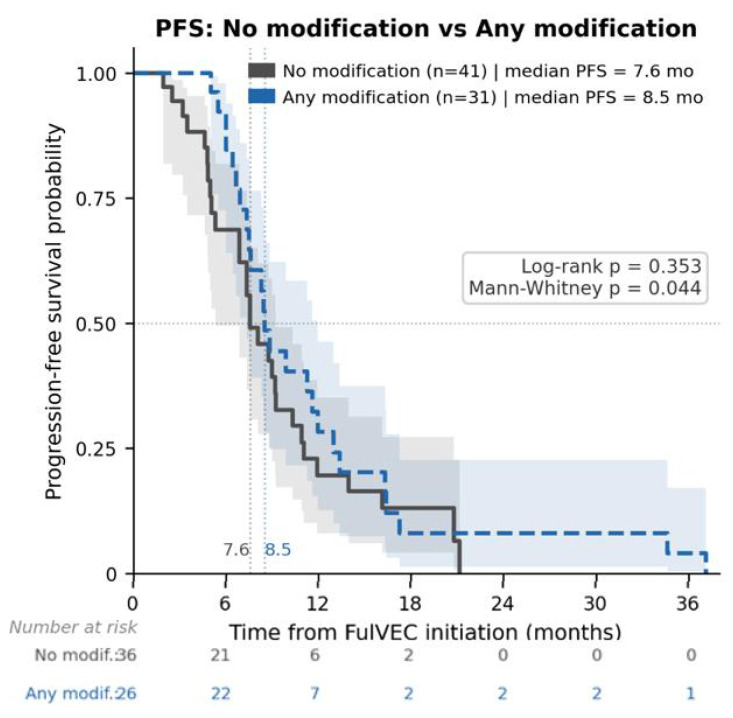
Kaplan–Meier progression-free survival curves for patients with any dose modification (dose reduction and/or treatment delay; n = 31) versus no modification required (n = 41). Median PFS: 8.5 months versus 7.6 months; log-rank *p* = 0.353; Mann–Whitney *p* = 0.044. Shaded areas represent 95% confidence intervals.

A complementary, purely descriptive perspective is provided by the dichotomized analysis (Figure 4). Although the log-rank test did not reach significance (*p* = 0.353) and the comparison is subject to the same confounding considerations discussed above (treatment duration and survivor bias), the modified-dose group showed a longer tail of durable disease control beyond 20 months than the no-modification group. We present this as a hypothesis-generating observation only, not as evidence of a treatment effect.

Several limitations require explicit acknowledgment. First, as discussed above, immortal time bias and confounding by treatment duration cannot be fully excluded and remain a plausible partial alternative to the pharmacological exposure hypothesis. Second, RECIST-based response data are unavailable for most patients, reflecting the retrospective design and the intentional minimization of CT/contrast burden during long-term palliative therapy; PFS, OS, and quality of life were considered the most clinically meaningful endpoints in this context. Third, the dose–response analysis is post hoc and hypothesis-generating, with an inherent risk of overfitting and multiple comparison inflation, and none of the between-group PFS or OS comparisons reached statistical significance. Fourth, granular, CTCAE-graded, event-level toxicity data (e.g., by specific AE type and severity grade) were not consistently available in this six-year retrospective dataset; only the resulting clinical intervention (dose reduction or treatment delay) and its broadly attributed cause were documented, which limits the precision of our safety characterization and prevents formal grade-specific safety reporting. Fifth, specific component-level modification data (i.e., which individual drug was reduced or delayed) were unavailable, precluding drug-level analysis. Sixth, the treatment delay subgroup comprised only 12 patients, substantially limiting statistical power for this comparison. Finally, cross-study comparisons with PALOMA-3, PACE, and real-world post-CDK4/6i series are descriptive only, given differences in study design, prior treatment lines, patient selection, and endpoint definitions between these studies and the present cohort. Given these limitations, the dose–response findings reported here should be regarded as hypothesis-generating and exploratory; they do not, on their own, justify a change in clinical practice. A prospective, adequately powered study, ideally incorporating pharmacokinetic sampling, prespecified CTCAE-graded toxicity assessment by drug component, and a randomized comparison between fixed-dose and toxicity-guided adaptive dosing of FulVEC, with PFS as the primary endpoint, is needed to formally test the pharmacological exposure hypothesis proposed here.

## 5. Conclusions

This extended, real-world cohort of 72 patients confirms the reproducible activity and manageable toxicity of the FulVEC metronomic chemo-endocrine regimen in heavily pretreated metastatic ER+/HER2− breast cancer, including patients who had previously failed CDK4/6 inhibitors and/or fulvestrant, for whom therapeutic options remain limited. No patient required permanent treatment discontinuation due to toxicity, supporting the practical feasibility of this stepwise dose adjustment approach in routine clinical practice.

From a practical standpoint, these findings support the continued use of FulVEC as a treatment option in this difficult-to-treat, post-CDK4/6i population, and are consistent with its inclusion among available salvage regimens where access to newer agents (e.g., antibody–drug conjugates) is limited by cost or availability. However, the exploratory association between treatment-emergent AEs and efficacy outcomes reported here, while intriguing and biologically plausible, is derived from a retrospective, single-center cohort with important limitations, including immortal time bias, small subgroup sizes, and non-significant survival comparisons. We, therefore, consider this association hypothesis-generating rather than confirmatory, and it does not, at present, warrant any change to current dosing practice or clinical guidelines. Formal prospective testing, ideally within a randomized trial comparing fixed-dose versus toxicity-guided adaptive dosing of FulVEC, is required before this concept could inform clinical recommendations.

## Figures and Tables

**Table 1:** Patient characteristics (N = 72).

Parameter	N (%)	JCM 2023 (N = 38) [13]
Age, median (range), years	50 (31–79)	46 (30–80)
ECOG PS 0/1/2/3	12/44/10/5 (17/61/14/7%)	NR
Prior systemic lines, median	2 (range 1–9)	2 (range 1–9)
≤2 lines	39 (54%)	20 (53%)
3–4 lines	18 (25%)	8 (21%)
≥5 lines	14 (19%)	10 (26%)
Prior CDK4/6 inhibitor	44 (62%)	18 (47%)
Prior fulvestrant	40 (56%)	20 (53%)
Before any VEC component	33 (46%)	30 (77%) *
Capecitabine	24 (34%)	13 (34%)
Vinorelbine	19 (27%)	11 (29%)
Cyclophosphamide	22 (31%)	12 (32%)
Bone metastases	57 (80%)	31 (82%)
Liver metastases	44 (62%)	25 (66%)
Lung metastases	25 (35%)	13 (34%)
CNS metastases	6 (8%)	3 (8%)
Organs involved, median (IQR)	3 (2–4)	NR

* JCM 2023 reported prior use of any FulVEC component (including fulvestrant); VEC-only exposure was 48.7%.

**Table 2:** Efficacy outcomes.

Endpoint	N = 72	JCM 2023 (N = 38) [13]
PFS, median months (95% CI)	8.5 (6.5–11.6)	8.4 (6.5–11.6)
PFS 12-month rate	23%	21%
PFS 24-month rate	5%	3%
OS, median months	18.0 (14.7–22.7)	21.5 (16.7–27.6)
OS 12-month rate	70%	63%
OS 24-month rate	31%	21%
Any CA15-3 decline, n/N (%)	31/38 (81.6%)	30/39 (76.9%)
≥50% CA15-3 decline, n/N (%)	17/38 (44.7%)	19/39 (48.7%)
(1–49% CA15-3 decline), n/N (%)	14/38 (36.8%)	11/39 (28.2%)
Any CA15-3 increase, n/N (%)	7/38 (18.4%)	9/39 (23.1%)

**Table 3:** Exploratory dose–response analysis: efficacy outcomes by AE-grade category.

Endpoint	Grade 0 No Modification (n = 41)	Grade 1 Dose Reduction (n = 19)	Grade 2 Treatment Delay (n = 12)	*p*-Value *
Non-progression, %	73.2	84.2	91.7	0.237
Biochemical benefit, %	68.4	90.9	100.0	0.07 †
CA15-3 change, median %	−34	−44	−52	0.07 †
PFS, median months	7.6	8.5	8.8	0.583
OS, median months	16.5	21.2	18.0	0.743
AE grade vs. treatment duration (Spearman r)	—	—	—	r = 0.258, *p* = 0.043

* Chi-square (non-progression; biochemical benefit) or Kruskal–Wallis (CA15-3 change; treatment duration); log-rank (PFS, OS). † trend.

**Table 4:** Safety outcomes.

Toxicity-Related Intervention	N = 72, % (n)	JCM 2023, % (n)
Any modification (dose reduction and/or delay)	43% (31)	NR
Dose reduction	41% (29)	46% (18)
Myelotoxicity-related	~80%	80%
Hand–foot syndrome-related	~15%	15%
Treatment delay	17% (12)	23% (9)
AE-attributable delay	13% (9)	NR
Permanent discontinuation due to AE	0% (0)	0% (0)

## Data Availability

The data are unavailable due to ethical restrictions.

## Abbreviations

The following abbreviations are used in this manuscript:

AE	Adverse Event
AI	Aromatase Inhibitor
CA15-3	Cancer Antigen 15-3
CDK4/6i	Cyclin-Dependent Kinase 4/6 Inhibitor
CET	Chemo-Endocrine Therapy
CI	Confidence Interval
CTCAE	Common Terminology Criteria for Adverse Events
ECOG	Eastern Cooperative Oncology Group
ER	Estrogen Receptor
ET	Endocrine Therapy
FulVEC	Fulvestrant + Vinorelbine, Endoxan (Cyclophosphamide), Capecitabine
HER2	Human Epidermal Growth Factor Receptor 2
HFS	Hand–Foot Syndrome
HR	Hazard Ratio
MCT	Metronomic Chemotherapy
MTD	Maximum Tolerated Dose
OS	Overall Survival
PD-1/PD-L1	Programmed Cell Death Protein 1/Programmed Death-Ligand 1
PFS	Progression-Free Survival
VEC	Vinorelbine, Endoxan (Cyclophosphamide), Capecitabine
